# Research on data transmission system based on expert library reinforcement learning in integrated network

**DOI:** 10.1371/journal.pone.0333372

**Published:** 2025-11-25

**Authors:** Ziyang Xing

**Affiliations:** Shanghai Donghai College, Shanghai, China; Beijing Institute of Technology, CHINA

## Abstract

With the continuous advancement of network transmission technology, more and more applications are being applied in wireless network environments, especially in places that require high coverage, such as oceans and mountainous areas. However, wireless data transmission has the disadvantages of unstable transmission and easy interruption using traditional methods. Based on this, we propose a data transmission system that uses a micro-electron-mechanical system (MEMS) sensor to obtain the wireless network status and applies expert library reinforcement learning that does not rely on reward functions to achieve retrieval enhancement of data transmission. Experimental verification shows that the proposed expert library reinforcement learning has strong generalizability and fast convergence.

Expert library reinforcement learning, wireless network, MEMS, integrated network.

## Introduction

**Fig 1:**
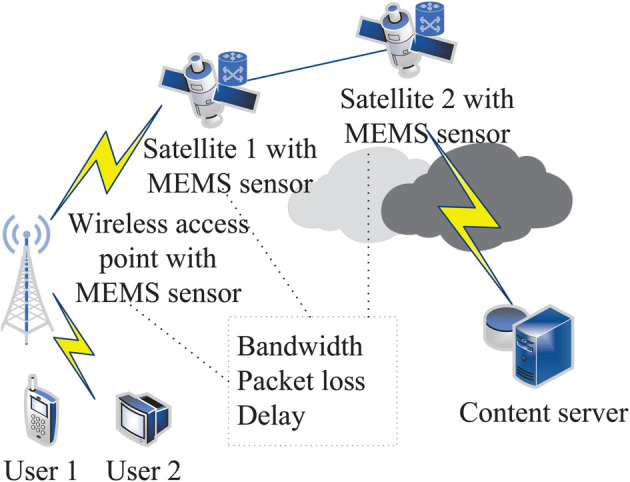
Data transmission scenario diagram in integrated network.

Wireless transmission refers to a method of data transmission using wireless technologies such as infrared and microwave. It has the characteristics of low cost and wide coverage and is widely used in the fields of Internet of vehicles, integrated space and earth networks, etc. In current Internet applications, the proportion of wireless data transmission has been reported to exceed that of data transmission in traditional wired networks [1]. However, in wireless networks, there are also shortcomings, such as large fluctuations in data transmission and insufficient bandwidth resources, which seriously affect normal data transmission and may cause a decrease in throughput or even communication failure [2]. In addition, in the process of traditional wireless network data transmission, when faced with emergency situations such as earthquakes and wars, there will be situations that affect data transmission, such as poor resource utilization and low path retrieval rate, and even a decrease in throughput and eventual transmission failure. The data transmission application scenario in wireless networks is shown in Fig 1. Ground users obtain content through ground base stations and low earth orbit satellites.

Therefore, in the current wireless data transmission process, there are the following problems that need to be solved.

1. In emergency situations such as earthquakes, due to poor link transmission and low bandwidth, data transmission cannot be achieved efficiently and stably.

2. In emergency situations, the demand for computing resources is growing exponentially at an unprecedented rate. Traditional large models cannot meet the current situation well, and the retrieval performance path retrieval rate is low and cannot meet data transmission.

MEMS is a technology that integrates micro-mechanical structures and electronic circuits on the chip. It combines microelectronics technology and can sense, control or perform physical actions. It is one of the core technologies in the fields of the Internet of Things and smart devices. So based on this, it can be applied to obtain certain parameters in the wireless network. MEMS technology can be combined with network testers, network traffic measurement equipment and other equipment to measure network transmission status, obtain and report to managers.

Artificial intelligence large models usually refer to models with parameter scales of tens of billions, hundreds of billions, or even trillions. Large language models refer to deep learning models trained with large amounts of text data, which enable the model to generate natural language text or understand the meaning of language text. The core idea is to learn the patterns and structures of natural language through large-scale unsupervised training and simulate the human language cognition and generation process. Large language models are mainly used in the field of natural language processing, aiming to understand, generate, and process human language text or error messages in machine malfunctions. These models can perform tasks including text generation, machine translation, recognition error,and sentiment analysis by training on large-scale text data [3]. Some scholars have applied large models to data transmission to solve problems such as the complexity of machine learning models and misunderstandings [4]. For example, Javaid *et al*. applied large language models in integrated networks to solve problems such as resource allocation and bandwidth setting [5]. Large language models can give the optimal strategy for the current state based on error information, previous transmission logs, etc., which is conducive to achieving the best action and avoiding disadvantages such as network congestion and load imbalance.

Expert library reinforcement learning is an artificial intelligence model that has gradually developed on the basis of traditional reinforcement learning and is not overly dependent on learning samples. It is good at solving decision-making problems such as weather forecasting and autonomous driving. Its principle is to solve the optimal strategy in a new environment based on previous expert strategies. It is very suitable for wireless networks with dynamic and unknown transmission environments. Reinforcement learning first generates expert strategies (standard answers) based on previous samples, and then evaluates the potential results of each action (standard answer scoring) based on the expert strategy, thereby outputting better optimization strategies for dynamic wireless network transmission decision problems.

The main contributions of this paper include:

1. Aiming at the problems of transmission interruption that may occur in wireless network data transmission, a reinforcement learning model based on expert library is proposed. The reinforcement learning based on expert library first uses MEMS to obtain the current state of the wireless network, and generates expert trajectories based on previous samples, and then generates strategy guidance for other inputs. Since the actions generated by reinforcement learning do not depend on the reward function, the generalization becomes stronger.

2. The proposed reinforcement learning model based on expert library is experimentally verified, which can solve the retrieval enhancement in wireless network transmission and has the characteristics of fast convergence.

Next, the design of the reinforcement learning model based on the expert library will be discussed in detail.

## 1 Related work

Wireless data transmission refers to a way of transmitting data using wireless technology, such as mobile phones accessing satellite Internet through base stations, satellites distributing remote sensing images to ground users, etc. It has the advantages of low cost, high coverage and flexibility and convenience. It has been widely used in forest fire prevention, earthquake emergency and other fields, and wireless data transmission is playing an increasingly important role. Wireless networks also have disadvantages such as dynamic changes and high packet loss rates, so it is necessary to comprehensively consider the characteristics of wireless networks and design efficient algorithms to improve transmission efficiency. With the rapid popularization of smart mobile devices, it is not possible to carry out artificial intelligence applications well. Han *et al*. redesigned the loss function to address this problem, and experiments showed that there are advantages in many aspects [6]. Zhang *et al*. proposed a scoring auxiliary framework to address the problem of affecting federated learning training in wireless medical Internet of Things, which is conducive to training better algorithms [7]. Tenllado *et al*. proposed a special protocol to improve transmission efficiency in response to the problem of poor data transmission caused by high packet loss rates in wireless networks [8]. Zhang *et al*. proposed a deep learning-based multiple access (DeepMA) method, which was experimentally proven to significantly improve bandwidth efficiency [9]. Huang *et al*. proposed a spatial prediction active transmission algorithm to actively estimate future positions and transmit data over long distances to address the problem of wireless networks affecting data transmission [10].

Verma *et al*. designed a MEMS sensor to obtain detailed physiological parameters of birds [11]. Sileo *et al*. used MEMS to detect temperature, relative humidity, and air quality [12]. Deepak *et al*. [13] used MEMS to obtain detailed transmission parameters in the Internet of Things. Therefore, it is feasible to use MEMS in wireless networks to obtain network status such as bandwidth, packet loss rate, and transmission delay.

Artificial intelligence big models usually refer to a type of artificial intelligence model with a large number of parameters built by artificial neural networks, which has the characteristics of strong generalization ability and strong learning ability. Big models have played an increasingly important role in decision-making, complex reasoning, target detection and other fields. For example, Alsadat *et al*. [14] designed a multi-agent reinforcement learning big model to integrate advanced knowledge. Ji *et al*. proposed a cloud edge large-micro model collaboration (CELTC) architecture based on deep reinforcement learning to optimize monitoring data processing and decision-making in offshore drilling platform scenarios [15]. Chen *et al*. applied reinforcement learning to obtain adaptive methods to learn optimal strategies and provide decisions for big model reasoning [16]. Wu *et al*. combined federated learning and multi-agent reinforcement learning to propose a big model that improves the utilization of computing resources [17]. Wang *et al*. focused on the application of big models to solve problems such as robot diversity [18]. McIntosh *et al*. observed the effectiveness of human feedback reinforcement learning (RLHF) on the initial answers of large language models and designed a big model to manipulate ideology [19].

From the above research status, it can be seen that it is completely feasible to solve the drawbacks of wireless network transmission by using the model designed based on expert libray reinforcement learning [20].

## 2 System modeling and implementation

### 2.1 Mathematical model

The entire data transmission system consists of ground users, space satellite constellations, content producers, etc. The function is to efficiently complete user requests and allocate resources. Include the following entities [21]:

Ground users: composed of a variety of terminals, request and obtain resources, such as the need to download 7:30 in a certain area of remote sensing images.

Ground wireless signal access point: connects ground users with aerial satellites and is equipped with MEMS sensors.

MEMS sensor: Because MEMS sensor has the function of data recording, it can record the number of data packets sent and received. Therefore, this paper proposes a MEMS sensor based on network protocol, which can capture and analyze data packets in the network, and can count the following functions [22]:

Bandwidth (*B*) is obtained by calculating the ratio of the number of bytes successfully transmitted to the time period. *Data*_*i*_ represents the amount of data.

$$B=\frac{Data_{2}-Data_{1}}{time_{2}-time_{1}}$$ (1)

The packet loss rate *C* is obtained by calculating the number of bytes successfully transmitted and the total number of bytes sent. *Data*_*total*_ represents the total of data. $Data_{receive}$ represents the total of data received successfully.

$$C=\frac{Data_{total}-Data_{receive}}{Data_{total}}$$ (2)

The time delay D is obtained by using an RTT between the transmit end and the receive end.

$$D=RTT_{2}-RTT_{1}$$ (3)

The above network parameters generated by the MEMS sensor can be used for path calculation, data scheduling, etc.

Satellite constellation: To expand the transmission of wireless networks, the satellites in the satellite constellation are equipped with MEMS sensors, which act as switches, and can obtain the remaining bandwidth, packet loss rate, delay, etc. in the link, which is recorded as: M=|(m|m=1,2,3,…,|M|)|, and the total number of satellites is recorded as: |M|. In order to avoid transmission congestion and improve satellite utilization, multiple satellite transmission links are used to transmit data as much as possible at the same transmission time. Affected by the periodic orbit switching of the satellite, the satellite will be available or unavailable, which is expressed as follows:

$$L(m)=\left\{ \begin{array}{c} 0,\text{ satellite }m\text{ is unavailable }\\ 1,\text{ satellite }m\text{ is available } \end{array}\right.$$ (4)

Content server: a variety of resources are stored, which can serve different ground users and respond to specific resources according to users’ requests. For example, a user requests remote sensing images of a certain area at 7:30. The content server can return specific remote sensing images through satellite constellations, wireless access points, etc.

Because the satellite has the characteristics of periodic dynamic change, the transmission link will be intermittent, so this paper divides a transmission process *T* into multiple time slots, assuming that the transmission link is stable and fixed in a time slot *t*. And there are:

$$T=\{t_{1},t_{2},t_{3},\ldots \}$$ (5)

The entire transmission is composed of a ground network, a space network, etc., and then the entire data transmission problem is modeled. Since the link formed by the selected satellite is the key information in the entire transmission process, we model the entire constellation into a three-dimensional coordinate system (*x*,*y*,*z*), where each satellite corresponds to a unique three-dimensional coordinate, for example, the coordinate value of the satellite numbered 12 is (34,98,100). Then in a satellite constellation, the Manhattan distance the satellite *m* and the satellite *n* is:

$$p(m,n)=|x_{m}-x_{n}|+|y_{m}-y_{n}|+|z_{m}-z_{n}|,\;where\;L(m)=0,L(n)=0$$ (6)

Because the satellite distance directly determines the transmission time (data transmission speed is the speed of light, fixed value). Since the transmission quality between different links is different, the transmission cost *O* is proposed, the lower the value, the more conducive to the realization of efficient transmission, on the contrary, the higher the transmission cost, the less conducive to transmission, which is defined:

O∝{B,−C,−D} (7)

The above formula indicates that *O* is proportional to the bandwidth *B* and inversely proportional to the packet loss rate *S* and the delay *D*, and its specific value can be obtained from the experiment.

Where the path from the satellite *m* to the satellite *n* is $\hat{p(m,n)}$:

$$\hat{p(m,n)}=\{\langle I_{m},I_{1}\rangle ,\langle I_{1},I_{2}\rangle ,\ldots ,\langle I_{a-1},I_{a}\rangle ,\langle I_{a},I_{n}\rangle \}$$ (8)

Mixed integer programming is used to establish an optimization model for the optimal transmission path and selection of satellite nodes. By defining satellite selection variables as integer types, the model can accurately describe the discrete decision-making process.

Therefore, according to the transmission strategy, it is necessary to constantly solve for the minimum value of the transmission path multiplied by its cost, that is, the distance that is most conducive to efficient transmission, which belongs to the mixed integer planning (MIP) that is:

O∝{B,−S,−D} (9)

where the constraints of the above equation are as follows:

(1) The selected satellite should be greater than 1.

a>1,a∈M (10)

(2) Only satellites within the constellation can be selected.

$$x_{min}\leq x_{a}\leq x_{max}\;y_{min}\leq y_{a}\leq y_{max}\;z_{min}\leq z_{a}\leq z_{max}$$ (11)

(3) The entire transmission link can meet the requirements of the amount of data transmitted between the user and the content server.

$$Data_{link}\approx Data_{user}\approx Data_{server}$$ (12)

where *Data*_*link*_ represents the total amount of data on the transmission link. *Data*_*user*_ represents the total number of requests the user needs to send. $Data_{server}$ the total quantity returned by the content server.

(4) The transmission time slot is continuous and continuously forward.

$$t_{1}\to t_{2}\to t_{3}\to \ldots$$ (13)

(5) MEMS sensor to obtain network state parameters can meet the needs of calculation.

$$\begin{array}{c} \sum b_{i}\geq |B|,i\in I\\ \sum c_{i}\geq |C|,i\in I\\ \sum d_{i}\geq |D|,i\in I \end{array}$$ (14)

When solving multi-objective optimization, it is impossible for each objective to reach the optimum at the same time, and each objective must have its own weight. We use the weight method to convert it into single objective optimization.

### 2.2 Expert library reinforcement learning

Due to the limited computing and storage capacity of satellites, in order to speed up the convergence speed of artificial intelligence algorithms, this paper proposes a dynamic experience playback mechanism, which can not only scatter the data to clear the correlation. Due to the fact that the selection of transmission nodes will affect the next action, it is a Markov decision process (MDP).

State set: The network state of nodes in the transmission path.

Actions: Which node should be selected as the transmission path.

Transition Probability: Describes the probability of transitioning from one state to another after taking a certain action.

Reward Function: Experts learn from historical logs, generate expert trajectories, and finally form expert strategies.

Set a training data tuple trasition:(RL) and $trasition:(RL^{\prime })$ the latest tuple to store in a queue, called replay buffer, with a capacity of *k*. Under a time slot *t*, it can store up to $\max_{t}trasition(RL)=k$.

When the replay buffer queue is full, every time a new one $trasition(RL^{\prime })$ is stored, that is, the oldest *trasition*(*RL*) is deleted, which is not conducive to the application of the integrated network containing satellites, based on this, we propose a dynamic experience playback pool, which speeds up the speed of data exchange and is conducive to rapid convergence. That is, in the training data tuple, the current experience playback pool of data total:

$$trasition(RL,k_{total})$$ (15)

Among them, is the number of existing experience playback, when $k\approx k_{total}$, directly join the experience playback pool, otherwise need to break up and delete the previous training data. This mechanism facilitates efficient utilization of training data.

**Fig 2:**
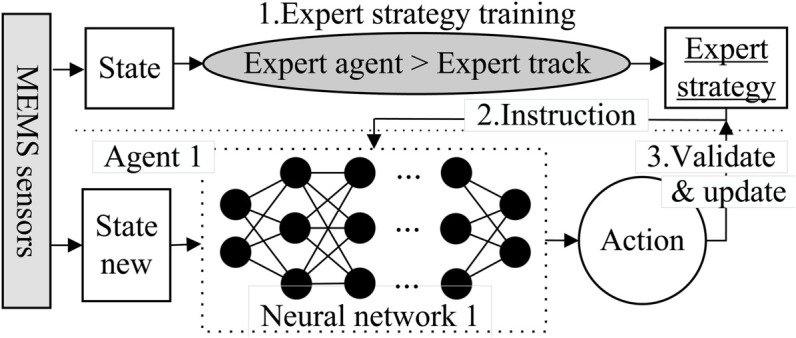
Principle of expert library reinforcement learning designed.

As shown in Fig 2, the state is obtained from MEMS and user input. Expert library reinforcement learning is divided into two parts: expert strategy learning and application reasoning. The expert strategy learning part mainly summarizes and learns expert strategies based on previous historical experience and stores them in the expert strategy library, which can provide guidance for the application reasoning of the second part. The second part includes state input, neural network (feature analysis of the input state, including input layer, hidden layer and output layer), and action output. The biggest difference between its application reasoning and traditional reinforcement learning is that it does not use reward function, but uses expert strategy library. Its advantage is that the adaptability range becomes wider and the generalization ability becomes stronger.

The workflow of expert library-based reinforcement learning is divided into:

- The expert agent generates expert trajectories based on the input, saves them as expert strategies by type, and finally stores them in the expert strategy library. For example: The action in the state $S_{1}^{E}$ is $A_{1}^{S_{1}}$, then its expert trajectory is identified as $S_{1}^{E}\to A_{1}$, and then, the expert strategy library under a certain category is counted as: $\Phi_{E}:S_{1}^{E}\to A_{1}$, where $\Phi_{E}$ is the category number.
- When the reinforcement learning agent encounters other inputs $D(S_{NEW},A_{New})$, it reasoned based on the expert strategy and generated the optimal action for a certain state, such as $\{\Phi_{E}:S_{1}^{E}\to A_{1}\}\to 1$.
- According to the generated optimal action, the conclusion is verified and the status of the expert strategy library is updated $\left\{\Phi_{New}\}\;\cup \;\{\Phi_{E}:S_{1}^{E}\to A_{1}\right\}$. In the whole process, the function used for comparison and verification is:
  $$max\{D_{NEW},max\{\Phi_{E}\}\}$$ (16)

The meaning is to first find the largest value from the expert strategy, and then compare the smallest difference with the input value. The above analysis uses the PPO (Proximal Policy Optimization) algorithm to solve the expert library problem. PPO is a reinforcement learning algorithm based on the Actor-Critic architecture. It achieves stable training by limiting the policy update amplitude. The core innovation is the introduction of the clipped surrogate objective. The algorithm has some advantages of Policy Gradient and Trust Region Policy Optimization. It imposes restrictions in the objective function to ensure that the difference between the new and old parameters is not too large. It alternates between sampling data and using the stochastic gradient ascent method to optimize the replacement objective function. PPO ensures training stability through pruning strategy updates, making it more stable and suitable for complex high-dimensional environments. Although the standard policy gradient method performs a gradient update for each data sample, PPO proposes a new objective function that can achieve small batch updates.

The core of the PPO algorithm [23,24] is to limit the magnitude of policy changes during policy updates, to avoid a sharp decline in policy performance caused by excessive updates. This is like the gearbox of a car, which needs to gradually increase from low gear to high gear from initial to official driving. If it is directly engaged in high gear, the engine can suffocate. See Algorithm 1 for more details.

**Algorithm 1: Reinforcement learning algorithm based on PPO.**

**Input:** Values to be compared *D*_*NEW*_, expert strategy library $\left\{\Phi_{E}\right\}$.

**Output:** An optimal action.

1: Repeat

2:   Repeat

3:    Get the right policy *π* with the state *s*.

4:    Create all the action with *D*_*NEW*_,like $\left\{A_{D1},A_{D2},A_{D3},\ldots \right\}$

5:    The loss function is: $\arg\min\sum_{actor}|V_{new}(\pi ;s)-V_{old}(\pi ;s)+\tau |$.

6:    *V* is the value in this iteration step.

7:    $V_{old}$ refers to the value of data collection.

8:    Perform gradient calculation: $\nabla O=𝔼[\sum (\nabla_{\theta }log(\frac{\pi (\theta ;s)}{\left(\pi_{old}(\pi_{old};s)\right)})\cdot A_{D})+log(\frac{s}{s_{old}})]$.

9:    𝔼 is expected calculation.

10:   Until (actor=1,2,3,…)

11:   Update policy $\pi_{E},\theta$ and $\left\{\Phi_{E}\right\}$.

12: Until (iteration=1,2,3,…)

In the above algorithm, line 5 indicates iterative comparison of the input value with the value in the expert strategy library and solving it with gradient. PPO usually updates the same batch of data multiple times to improve data utilization. *τ* is a very small number to prevent updates from happening too quickly.

### 2.3 Parameter fine-tuning

Fine-tuning is a secondary training for tasks such as wireless network data transmission based on the existing expert library-based reinforcement learning model. This technology uses small-scale, labeled data sets to adjust model parameters to better adapt to and accurately complete the decision-making of data transmission in wireless networks, such as which transmission link to choose when there are multiple paths. In this section, the open source library HF-PEFT framework developed by Hugging Face is used for fine-tuning.

The task is achieved by setting task requirements, determining fine-tuning strategies, defining loss functions, and optimizers. The best result is selected using random forests. Random forests use decision trees as weak classifiers. On the basis of random sampling of bagging samples, random selection of features is added . Random forest is a compositional supervised learning method. In random forests, we generate multiple prediction models at the same time and summarize the results of the models to improve the accuracy of the prediction models.

The method of establishing a decision tree: when the current number of features is *ρ*, randomly select a subset of features, and then select the optimal features for division, controlling the degree of randomness *ρ*, and having:

$$log_{2}\rho +\sigma$$ (17)

*σ* is a very small number used to adjust the value of an item, and the specific value can be obtained from the implementation.

We know higher the accuracy of a single decision tree, the higher the accuracy of the random forest. The algorithm implementation process for establishing a random forest is shown in Algorithm 2.

In this way, the model can learn the “probabilistic” expression of certain causal relationships through training with a large amount of data. For example, after the model “sees” the scene of “traffic accidents are more likely to occur on snowy days” countless times , it will also guess that there is a great correlation between slippery roads and traffic accidents.

**Algorithm 2: Fine-tuning the random forest algorithm.**

**Input:** Multiple classification results.

**Output:** The most important value.

1: Randomly select a sample from the sample set by placing it back into random sampling.

2: Calculate its recommended value with the Formula (17).

3: Build a decision tree using these features for the selected samples.

4: Repeat the above two steps to generate a decision tree and form a random forest, where the generated decision tree is not pruned.

The above algorithm has the advantages of low overhead, low complexity, easy implementation, and small differences between multiple individual learners.

## 3 Performance evaluation

The comprehensive data transmission performance of the proposed method, a model based on expert library reinforcement learning is designed with pytourch (https://pytorch.org/). The HF-PEFT framework is installed in the environment, mainly the Transformers library of Hugging face to implement the large model. The simulation parameters in the experimental evaluation are shown in Table 1. The satellite constellation (LEO,Low Earth Orbit) is the Iridium NEXT constellation.

**Table 1:** The simulation parameters.

LEO height (km)	780
Number of users	17
Bandwidth between satellite and ground (KMbps)	58
Loss	0.08
The content provider Service minutes	15

The algorithms compared in the experiment include:

Proposed method : refers to the method of strengthening the expert library proposed in this paper.

DRL: refers to the deep reinforcement learning algorithm in the Internet of Vehicles environment (without expert library), which solves the problem of weak generalization. For details, see the paper [25].

LLM: refers to an algorithm based on a large language model and reinforcement learning to solve reasoning problems such as resource allocation. For details, see the paper [26].

LsiA3CS: refers to the algorithm based on Actor-Critic reinforcement learning in the Internet of Things, which is used to solve task height problems. For details, see the paper [27].

GPT: refers to the algorithm based on reinforcement learning and large models in power networks. For details, see the paper [28].

### 3.1 Throughput normalization

**Fig 3:**
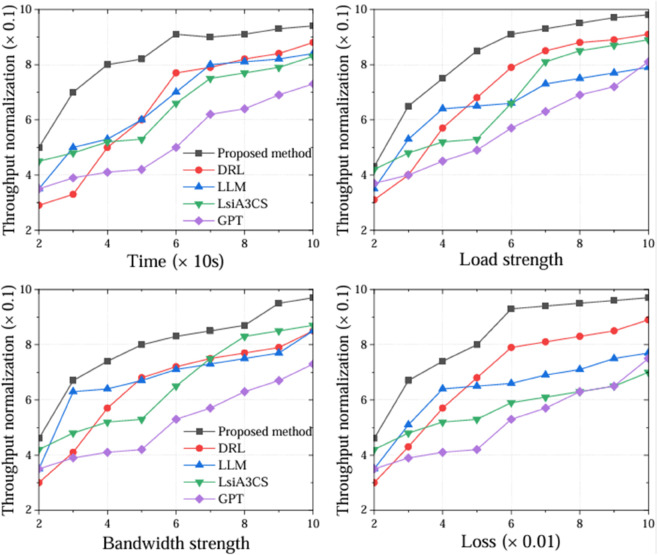
Throughput normalization comparison of several algorithms under different transmission conditions.

Throughput refers to the ratio of the amount of successfully transmitted data to the time under the same data transmission environment. There are many factors that affect throughput, such as network congestion, packet loss rate, etc. Since the proposed method algorithm is based on reinforcement learning of the expert library, it can adapt to data transmission in dynamic networks and can deal with problems such as network congestion, and has a higher advantage. DRL is an ordinary reinforcement learning algorithm that cannot cope with data transmission well and has large jitter. LLM and LsiA3CS also cannot cope with the dynamics of wireless transmission well, and transmission jitter also occurs. GPT has the function of reasoning. As shown in Fig 3, we conducted experiments from four aspects: transmission time, load strength, different packet loss rates, and bandwidth strength, and recorded the start (end) time and the amount of successfully transmitted data. The algorithm proposed has the advantages of intelligence and low time complexity, resulting in the highest throughput.

### 3.2 Normalization of algorithm convergence

**Fig 4:**
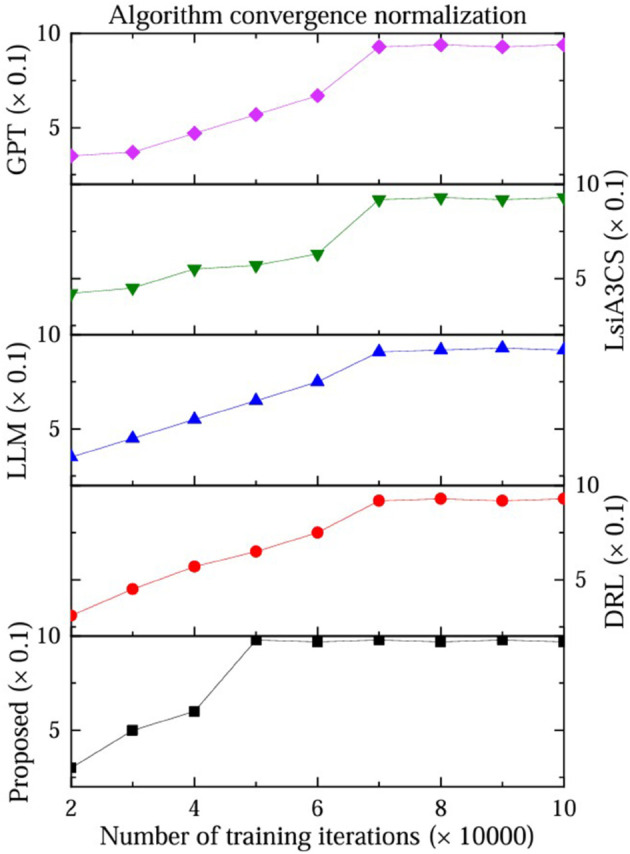
Convergence normalization comparison.

Convergence analysis is a mathematical method to study the speed and properties of the target algorithm approaching the minimum value during the iteration process. It is widely used in optimization algorithm design and performance evaluation. The core is to quantify the change law of the difference between the function value and the optimal solution with the number of iterations, and analyze the convergence speed and stability through different mathematical tools. The proposed method algorithm uses linear regression programming to solve the problem, so it has the advantages of low complexity and fast convergence. DRL is a reinforcement learning algorithm that relies on the reward function, so the convergence time is slow, as shown in Fig 4.

## 4 Conclusion

We propose a reinforcement learning framework based on an expert library to optimize data transmission in a dynamic environment. Specifically, We use MEMS to obtain the transmission state in the wireless network, models the optimization problem as a Markov decision process, generates expert strategies, and finally introduces a reward agent model to significantly shorten training time. Experimental results show that the proposed framework has the advantages of low complexity, is intelligent and affectionate, and can efficiently balance the reasoning performance and the computational overhead of the user end. It can be widely used in base station-based data networks, vehicle networks, and marine networks.

In future work, the generalization of the proposed framework needs to be further studied, and experiments will be carried out in larger-scale and complex astronomical networks. In addition, the steps and efficiency of generating expert strategies by imitation learning need to be further optimized.
